# Dynamics of Acute Local Inflammatory Response after Autologous Transplantation of Muscle-Derived Cells into the Skeletal Muscle

**DOI:** 10.1155/2014/482352

**Published:** 2014-08-27

**Authors:** Anna Burdzinska, Kamila Gala, Cezary Kowalewski, Radosław Zagozdzon, Zdzisław Gajewski, Leszek Pączek

**Affiliations:** ^1^Department of Immunology, Transplant Medicine and Internal Diseases, Transplantation Institute, Medical University of Warsaw, Nowogrodzka 59, 02-006 Warsaw, Poland; ^2^Department of Dermatology and Immunodermatology, Medical University of Warsaw, Koszykowa 82A, 02-008 Warsaw, Poland; ^3^Department of Immunology, Center of Biostructure Research, Medical University of Warsaw, Banacha 1A, 02-097 Warsaw, Poland; ^4^Department of Large Animal Diseases with Clinic, Warsaw University of Life Sciences (SGGW), Nowoursynowska 100, 02-797 Warsaw, Poland

## Abstract

The vast majority of myoblasts transplanted into the skeletal muscle die within the first week after injection. Inflammatory response to the intramuscular cell transfer was studied in allogeneic but not in autologous model. The aim of this study was to evaluate immune reaction to autotransplantation of myogenic cells and to assess its dynamics within the first week after injection. Muscle-derived cells or medium alone was injected into the intact skeletal muscles in autologous model. Tissue samples were collected 1, 3, and 7 days after the procedure. Our analysis revealed the peak increase of the gene expression of all evaluated cytokines (*Il-1α, Il-1* *β*, Il-6, Tgf-*β*, and *Tnf-α*) at day 1. The mRNA level of analyzed cytokines normalized in subsequent time points. The increase of *Il-* *β* gene expression was further confirmed at the protein level. Analysis of the tissue sections revealed rapid infiltration of injected cell clusters with neutrophils and macrophages. The inflammatory infiltration was almost completely resolved at day 7. The survived cells were able to participate in the muscle regeneration process. Presented results demonstrate that autotransplanted muscle-derived cells induce classical early immune reaction in the site of injection which may contribute to cellular graft elimination.

## 1. Introduction

Adult muscle tissue is highly exposed to the damage by both internal and external factors throughout the life span and therefore requires the ability to effectively regenerate. The cells which are primarily responsible for skeletal muscle tissue restoration capacity are called satellite cells. The idea to deliver myoblasts which are satellite cells progeny into the dysfunctional muscles as a method of treatment was presented for the first time in 1978 by Partridge and colleagues [1]. Since then myoblasts transfer therapy (MTT) has been a subject of extensive studies. At the beginning, myoblasts have been considered as a very promising alternative for treatment of muscular dystrophies (MDs); however it became clear that this population is not able to engraft skeletal muscle after systemic delivery [2]. The poor migratory capabilities hinder the potential use of myoblast-based therapy in Duchenne MD and narrow the possible applications to the disorders with more focal character. Nevertheless, myoblasts transfer is still perceived as possible future alternative treatment in numerous conditions. The list includes sphincters dysfunction (urethral, anal, esophageal, and pyloric), atrophy of reinnervated muscle, rectovaginal fistulas, local muscle loss due to injury, and some types of muscular dystrophies (like oculopharyngeal or facioscapulohumeral MDs). Unfortunately, myoblasts transfer procedure is associated with another crucial unsolved problem, the poor survival rate of donor cells. It was demonstrated that the vast majority of graft is lost within first 3 days after transplantation. The massive elimination of injected myoblasts was identified in 1996 by Fan and colleagues [3] and subsequently confirmed by many other studies. This phenomenon was observed regardless of type of animal model (murine or porcine), type of target area (skeletal muscle, myocardium, or urethral sphincter), status of host muscle (intact or injured), or status of the host organism (immunodeficient or immunocompetent) [4–9]. Moreover, Holzer and colleagues demonstrated that myoblasts are eliminated even after autologous transplantation [7]. Several potential causes of rapid myogenic cell death after transfer have been proposed. They include inflammatory response in the injection site [5], ischemia [10, 11], hypoxia, [12], and anoikis [13]. The immune reaction after myoblasts injection has been studied intensively in allogeneic models [5, 14–17] but not after autologous transplantation as this type of transfer is believed to be nonimmunogenic. However, our previous study demonstrated that oxidative stress might play a role in elimination of myogenic cells after autologous transplantation [18]. Therefore, we hypothesized that autologous transfer of myogenic cells also triggers early immune response associated with oxidative burst. As autologous transplantation is considered to be the safest option in all clinical applications listed above, it seems to be of prime importance to evaluate the local tissue response for this kind of grafting. Therefore, the aim of this study was to examine the presence and dynamics of cytokines expression and cells associated with innate immune reaction within first week after autologous MTT.

## 2. Materials and Methods

### 2.1. Animals

All experiments were performed on 3-month-old Lewis rats (in-bred strain). Animals were housed with free access to food and water and were maintained at a constant temperature. Animal housing and experimental procedures were approved by Local Ethics Animal Welfare Commission of the Medical University of Warsaw.

### 2.2. Isolation and Cultivation of Muscle-Derived Cells (MDCs)

Skeletal muscle samples (about 0.05 g) for cell isolation were obtained from* musculus gracilis* during general anesthesia induced by administration of xylazine (10 mg/kg; Leciva, Prague, Czech Republic), ketamine (40 mg/kg; Spofa, Prague, Czech Republic), and butorphanol (1 mg/kg; Fort Dodge Animal Health, Fort Dodge, IA, USA). Tissue sampling did not impair rats mobility after surgery. Isolation of muscle-derived cells was performed as described by Burdzińska et al. [19]. The cells were suspended in standard growth medium (GM), DMEM supplemented with 10% (v/v) fetal bovine serum and antibiotic, antimycotic mixture (all components purchased from Invitrogen, Carlsbad, CA, USA). In order to reduce number of fibroblasts in culture, the medium containing nonadherent cells was removed to another dish 24 h after cell seeding (preplating). The first change of culture medium was performed 72 h after isolation. When the culture reached 70% of confluence, cells were harvested by trypsinization (0.25% trypsin and 0.02% EDTA; Invitrogen-Gibco Carlsbad, USA) and reseeded in new dishes in a density of 5 × 10^3^/cm^2^. Majority of cells were cultured for transplantation whereas part of population were seeded separately to perform* in vitro* characterization, desmin expression and differentiation potential analysis.

### 2.3. Immunocytofluorescence and Differentiation Potential

To identify isolated cells, MDCs were analyzed for the presence of desmin, myogenic cells marker. Cells after the first passage were cultured in a Lab-Tek 4-chamber slide w/Cover (Permanox Slide Sterile, Nalge Nunc International, Naperville, IL, USA) until they reached 80% confluence; then they were fixed in 4% (w/v) paraformaldehyde for 15 min at room temperature and permeabilized with 70% cold methanol for 20 min in −20°C. Samples were treated with blocking solution (1% bovine serum albumin/5% normal donkey serum in phosphate-buffered saline) for 30 min in RT and then probed with mouse anti-desmin (Sigma-Aldrich, St. Louis, MO, USA, 1 : 50 v/v, 90 min, RT). Afterwards, cells were washed and probed with a secondary antibody [Alexa-Fluor 594 donkey anti-mouse (Jackson ImmunoResearch Europe, Suffolk, UK), 1 : 100 v/v, 60 min, RT]. Cells were visualized using fluorescent microscopy via Olympus IX51.

To verify myogenic potential, the other subsets of isolated cells were induced to differentiate by cultivation in DMEM supplemented with 2% of horse serum (HS) for 3 days. The differentiated cells were immunostained for desmin as described above. The fusion index was determined as the ratio of nuclei in myotubes to the total number of nuclei in the same field calculated from at least 10 fields of view per animal and was expressed as a percentage (0% to 100%). The presence of intracellular lipid droplets in MDC population was confirmed with Oil Red O staining (Sigma-Aldrich, St. Louis, MO, USA).

### 2.4. Cell Suspension or Vehicle Injection

For injection procedure rats were sedated with xylazine/ketamine mixture. The skin in the area of injection was shaved and disinfected. In the transplanted animals, MDCs suspended in 200 *μ*L DMEM were administered into the gastrocnemius muscle in autologous manner. Cell suspension was given through 22 G needle in a single bolus directly to the intact muscle without any skin incision. Sham animals were treated in the same way but received 200 *μ*L DMEM vehicle only. At the same time, additional 100 *μ*L of cell suspensions was directed for microbiological tests (bacteria and yeast). The injected cells were either unlabeled (animals designated for gene and protein expression analyses) or labeled with fluorescent membrane linkers, PKH26 (red dye, animals designated for immunohistochemical staining) or DiD (DiIC18(5)-DS [1,1-Dioctadecyl-3,3,3,3-tetramethylindodicarbocyanine-5,5-disulfonic acid] in animals for* in vivo* imaging). Before preparing the final suspension, the cells were washed twice in DMEM to remove serum completely.

### 2.5. Tissue Collection

The tissue surrounding the area of either cells or DMEM administration was harvested at day 1 (24 hours), day 3, or day 7 after the transplantation. In the untreated group, the analogous muscle fragments were collected. The tissue samples were immediately snap-frozen in liquid nitrogen and stored in −80°C until analysis.

### 2.6. RNA Isolation, Reverse Transcription, and Real-Time PCR Analysis

The animals designated for gene and protein expression analysis were transplanted with equal amount (1 × 10^6^) of cells (*n* = 18, 6 in each time point). MDCs for these experiments were unlabeled to avoid additional manipulations which are always associated with increased risk of acquired immunogenicity. Untreated (*n* = 7) and sham operated groups (*n* = 18, 6 in each time point) served as controls. Tissue samples collected at days 1, 3, and 7 were homogenized in TissueLyser homogenizer (Qiagen, GmbH, Hilden, Germany) at a frequency of 25 Hz for 5 minutes. Total RNA was isolated using RNeasy Fibrous Tissue Mini Kit (Qiagen, GmbH, Hilden, Germany). RNA concentration was quantified by spectrophotometer at 260 nm using NanoDrop (ND-1000 Spectrophotometer, NanoDrop Technologies, Inc.). Reverse transcription of total mRNA into cDNA was performed using the SuperScript III (Invitrogen, Gibco, Carlsbad, USA) according to the manufacturer's instruction. Real-time PCR was performed on ABI Prism 7500 Sequence Detector (Applied Biosystems, Foster City, USA). Specific primers and probes set were purchased from Applied Biosystems:* Il-1α* (Rn0055700_m1),* Il-1* *β* (Rn00580432_m1),* Il-6* (Rn00561420_m1),* Tgf-* *β* *1* (Rn00572010_m1), and* Tnf-α* (Rn01525859_m1).* Gapdh* gene (4352338E) was used for normalization. The values are expressed relatively to a reference sample (calibrator): not treated muscle. The Ct (threshold cycle) for the target gene and the Ct for the internal control were determined for each sample. The relative gene expression was calculated by 2^−ΔΔCt^ method.

### 2.7. ELISA

The evaluation of *Il-1α* and *Il-1* *β* concentration in tissue homogenates was performed by ELISA. The muscle samples were homogenized in a buffer with phosphates and proteases inhibitors (Sigma-Aldrich, St. Louis, USA). Then probes were clarified by centrifugation at 10 000 rpm for 5 minutes and addition of PMSF. Total protein concentration was measured using NanoDrop (ND-1000 Spectrophotometer). Cytokines concentrations in tissue lysates were determined using commercial available ELISA kits (R&D System, Minneapolis, MN, USA). The results were presented as an absolute ratio: interleukin concentration/protein concentration (×10^−9^).

### 2.8. Histology and Immunohistochemistry

To visualize injected cells in the host tissue cells were labeled with red fluorescent membrane linker, 5 *μ*M PKH26 (*n* = 6, 2 in each time point). The contralateral gastrocnemius muscles in this group were injected with vehicle only. Histological and immunohistochemical staining were performed on frozen sections (10 *μ*m thick) prepared with the use of cryostat Microm HM 525 (Microm, Walldorf, Germany). Some sections were stained with hematoxylin and eosin. Immunohistochemical staining was performed using primary antibodies against antigens: CD43 (1 : 20 v/v) and CD68 (1 : 20 v/v) (AbD Serotec, Kidlington, UK). Samples were fixed with cold acetone. Nonspecific binding sites were blocked with 5% normal donkey serum in PBS. Tissue sections were incubated with primary antibodies for 1 h at RT. Afterwards, cells were washed and probed with a secondary antibody [Alexa-Fluor 488 donkey anti-mouse] (Jackson ImmunoResearch Europe, Suffolk, UK, 1 : 100 v/v) for 1 h at RT. Finally, the slides were washed and covered with VECTASHIELD Mounting Medium with DAPI (Vector Laboratories LTD., Peterborough, UK). The samples were evaluated with Eclipse Ni-U microscope (Nikon, Tokyo, Japan).

### 2.9. *In Vivo* Imaging

For optical imaging an average of 3.3 × 10^5^ of MDCs were injected as described above (*n* = 3). The cells were labeled prior to the transplantation with 7.5 *μ*M DiD, a membrane linker with Ex-max 650 nm/Em-max 670 nm (AAT Bioquest, Sunnyvale, CA). The area of imaging was carefully shaved and the adjacent part of the body was covered with dark fabric to avoid hair-derived autofluorescence.* In vivo* imaging was carried out using the IVIS Spectrum system (Caliper Life Sciences, Hopkinton, MA). Automatic algorithm for spectral unmixing of DiD dye against the food and autofluorescence backgrounds was used for visualizing the transplant-specific fluorescent signal, with excitation wavelength of 640 nm and six emission wavelengths (680, 700, 720, 740, 760, and 780 nm). Imaging data were analyzed using Living Image 4.4 software (Caliper).

### 2.10. Statistical Analysis

Results from RT-PCR were presented as a fold change of gene expression in relation to the calibrator, whereas data from ELISA were expressed as means (SD). Results were analyzed in pairs (untreated control versus VEH group and VEH versus MDC groups in certain time points) using nonparametric* U* Mann-Whitney test. A value of *P* < 0.05 was considered as statistically significant. For RT-PCR assay the significance of differences between groups was measured in ΔCt values. All RT-PCR and ELISA analysis were done in duplicate.

## 3. Results

### 3.1. The Isolated Muscle-Derived Population Is Heterogeneous but Consists Primarily of Myoblasts

**Figure 1:**
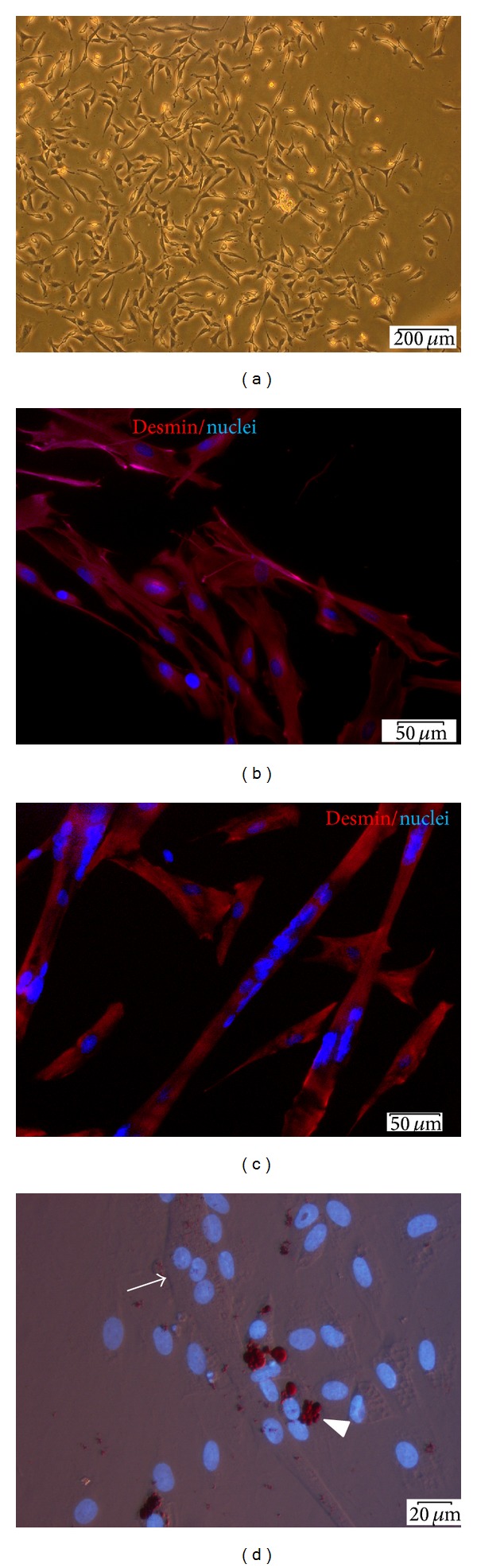
Identification of isolated muscle-derived cells. (a) The appearance of muscle-derived colony 6 days after isolation; (b and c) fluorescence microscopy. Desmin protein stained with Alexa 594 conjugated Ab (red) in undifferentiated MDCs (b) and in MDCs induced to myogenic differentiation for 3 days (c); (d) the presence of lipid accumulating cells within MDC population, red lipid droplets indicated with white arrowhead (Oil Red O staining), and cell nuclei stained with DAPI (blue). A multinucleated myotube can be observed in the same field of view (arrow). Scale bars in (a) 200 *μ*m, (b and c) 50 *μ*m, and (d) 20 *μ*m.

Within first days after isolation procedure, the mononuclear, colony-forming spindle shaped cells could be observed (Figure 1(a)). The mean percentage of desmin expressing cells in obtained MDCs population after the first passage amounted to 77% (Figure 1(b)). Isolated MDCs cultured in DMEM/2% HS differentiated into myotubes (Figure 1(c)), which confirms their myogenic potential. The mean fusion index was 35%. However, some cells in culture spontaneously accumulated lipids, which was visualized by Oil Red O staining (Figure 1(d)). These cells were probably the progeny of fibro-/adipogenic progenitor cells residing in muscle tissue.

### 3.2. Administration of Vehicle Itself Induces Significant Upregulation of* Il-1α*,* Il-6*, and* Tgf-* *β* Genes Expression

**Figure 2:**
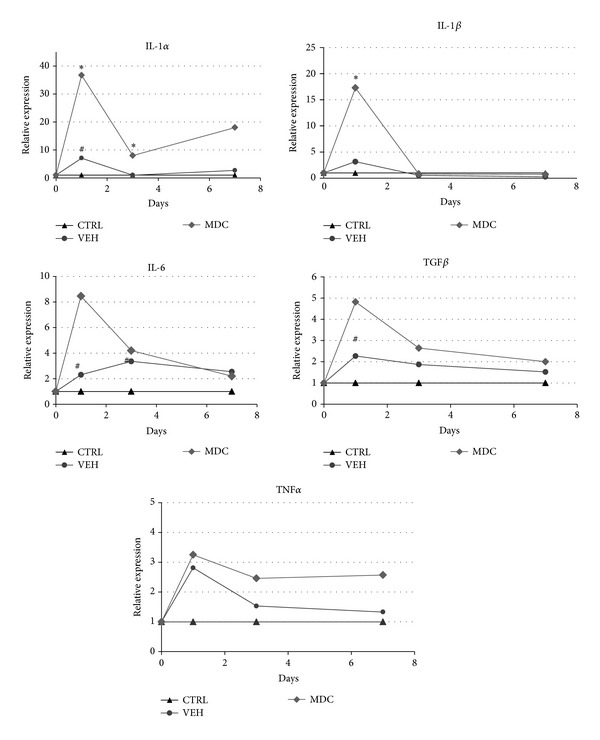
The analysis of cytokines gene expression in the muscle tissue determined by real-time PCR. The graphs represent relative gene expression of* Il-1α*,* Il-1* *β* ,* Il-6*,* Tgf-* *β* ,and* Tnf-α* in animals' muscles from different groups: untreated control (CTRL), after vehicle injection (VEH), and after MDCs transplantation (MDC) 1, 3, and 7 days after surgery. Results were analyzed in pairs (untreated control versus VEH and VEH versus MDC) using nonparametric* U* Mann-Whitney test. * indicates statistically significant difference between MDC and VEH groups and ^#^indicates statistically significant difference between VEH and CTRL groups in certain time points.

The analysis of gene expression revealed that needle insertion and administration of 200 *μ*L vehicle into the muscle caused significant upregulation of* Il-1α*,* Il-6*, and* Tgf-* *β* (7-fold, 2.3-fold, and 2.7-fold, resp.) at day 1 in the injection site comparing to the untreated control. The significant elevation of* Il-6* gene expression in VEH group comparing to CTRL group was maintained at day 3. The expression of other evaluated cytokines normalized at day 3. One week after the injection there was no statistical differences between VEH and CTRL groups (Figure 2).

### 3.3. Transplantation of Muscle-Derived Cells Causes Significant Upregulation of mRNA Level for* Il-α* and* Il-1* *β* Genes and Increases Il-1*β* Concentration at Day 1 after Injection in Comparison to the VEH Group

**Figure 3:**
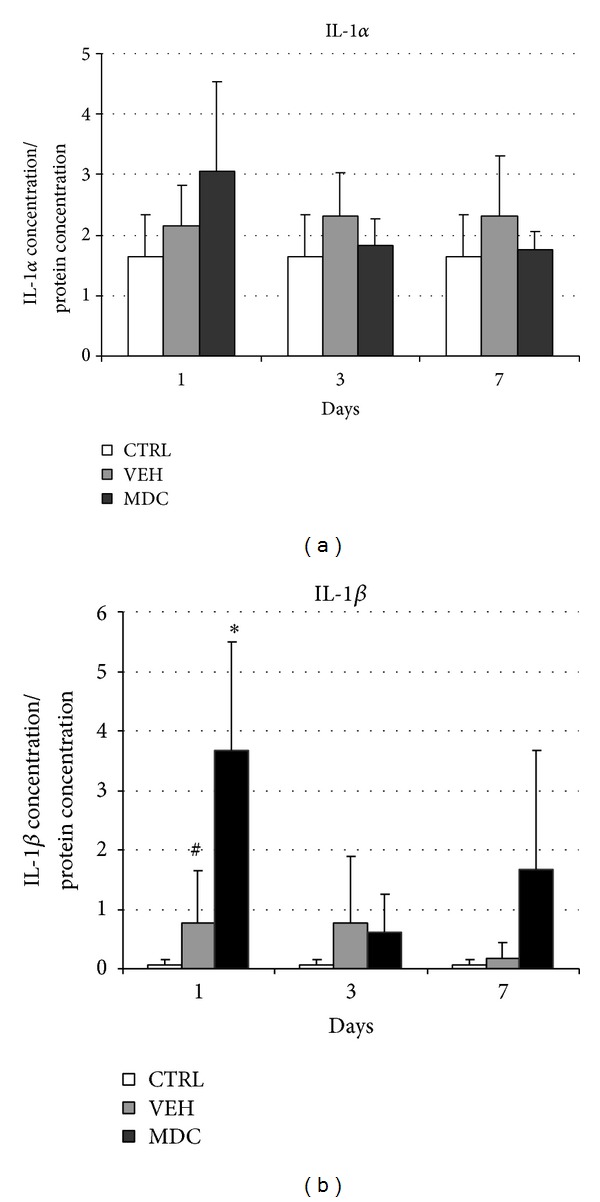
The concentration of cytokines: IL-1α (a), IL-1*β* (b) in the muscle tissue: untreated control (CTRL), after vehicle injection (VEH), and after MDCs transplantation (MDC) 1, 3, and 7 days after surgery. Protein concentrations of cytokines in tissue were determined using ELISA tests. Results were analyzed in pairs (untreated control versus VEH and VEH versus MDC) using nonparametric* U* Mann-Whitney test. * indicate statistically significant difference between MDC and VEH groups and ^#^indicate statistically significant difference between VEH and CTRL groups in certain time points.

Cell suspensions directed to transplantation were free from microbiological contamination. As the injection of vehicle itself caused significant differences in cytokines gene expression, the results obtained from transplanted samples were statistically analyzed in comparison to the sham control (VEH group). The administration of autologous cells caused significant increase in gene expression of* Il-1α* and* Il-1* *β* (Figure 2). The elevation was 5.5-fold and 5.2-fold, respectively, comparing to the VEH group and 36-fold and 17-fold, respectively, comparing to the untreated group. The expression of* Il-6*,* Tnf-α*, and* Tgf-* *β* displayed similar pattern (the elevation peak at day 1); however the differences in those cytokines were not statistically relevant. Significant changes in gene expression (both* Il-1α* and* Il-1* *β* ) were verified by evaluation of protein level in analogous samples. In the case of* Il-1α*, the protein concentration did not confirm upregulation of gene expression; the differences between groups were not statistically significant (Figure 3(a)). In contrast,* Il-1* *β* protein level strictly reflects transcriptional changes. MDCs administration induced significant 4-fold increase of proinflammatory* Il-1* *β* mean concentration in the site of injection 24 h after transplantation in comparison to the sham control (Figure 3(b)). At the same time, vehicle injection itself also caused significant local elevation of* Il-1* *β* level in comparison to the untreated control (Figure 3(b)).

### 3.4. There Are No Significant Differences in Cytokines Expression at Day 7 after MDC Autologous Transplantation

The dynamics of evaluated genes expression clearly demonstrate that upregulation of proinflammatory cytokines after autologous MDCs transfer is acute and transient. At day 3 only* Il-1α* expression was still significantly elevated in samples from MDC group in comparison to the sham group. At day 7 no significant differences in the mRNA level of examined cytokines were observed either after the transplantation or after the vehicle injection (Figure 2).

### 3.5. Distribution of Injected Cells Differs between Analyzed Time Points

**Figure 4:**
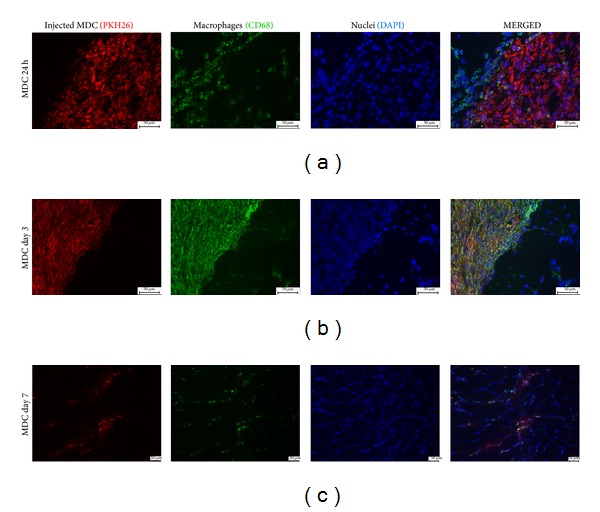
Immunohistochemical staining in different time points. Macrophages (CD68 antigen stained with Alexa 488 (green)) are infiltrating the cluster of transplanted MDC (PKH26 (red)). Cell nuclei stained with DAPI (blue). Scale bars: 50 *μ*m.

**Figure 5:**
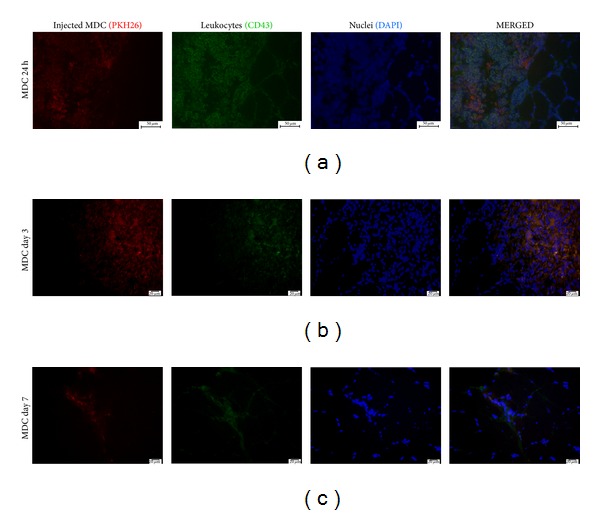
Immunohistochemical staining in different time points. Leukocytes (CD43 antigen stained with Alexa 488 (green)) are infiltrating the cluster of transplanted MDC (PKH26 (red)). Cell nuclei stained with DAPI (blue) in different time points. Scale bars: 50 *μ*m.

To identify injected cells at the injection site in some animals (*n* = 6) MDCs were labeled with red fluorochrome PKH26 prior to transplantation. The analysis of sectioned tissue samples revealed big, distinct clusters of PKH26 stained cells between muscle fibers (Figures 4 and 5). No red fluorescence was observed in sham operated limbs (data not shown). The transplanted cells formed dense clusters at day 1 and day 3 but not after one week after injection. PKH26 positive cells in tissue samples collected at day 7 were rather rare and diffused throughout the injection site.

### 3.6. Host Derived Cultured MDCs Induce Rapid Infiltration of Inflammatory Cells

**Figure 6:**
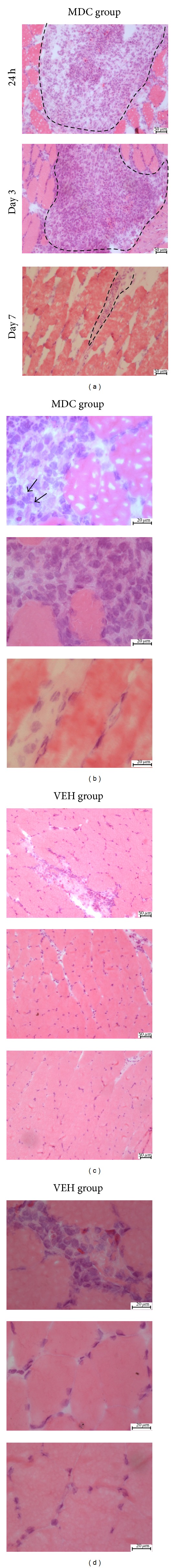
The skeletal muscle stained with hematoxylin and eosin. Images present the area of injection at days 1, 3, and 7 after procedure in subsequent rows. (a and b) Cross sections from transplanted group in two different magnifications. Distinct clusters of injected cells are visible. Dashed lines drawn on images in column A indicate approximate area of clusters located between muscle fibers. At day 1, neutrophils can be recognized within the cluster (arrows). At day 3, identification of neutrophils is not obvious; large mononuclear cells dominate in the cluster. At day 7, inflammatory infiltration is not clearly visible anymore; (c and d) cross sections from group injected with vehicle only in two different magnifications. Some inflammatory cells could be recognized in samples collected at day 1 time point.

To evaluate the presence of inflammatory cells in the injection site the tissue sections were stained with H&E (Figure 6). This preparation allowed for recognition of transplanted cell clusters in samples collected 1 and 3 days after injection. Numerous inflammatory cells, especially polymorphonuclear granulocytes, could be observed around and within the clusters at day 1. In specimens obtained 7 days after injection, inflammatory cells were not observed any more in relevant number. To characterize this infiltration better, the tissue sections were immunostained for CD68, macrophage marker, and CD43, leukosialin, present on majority of leukocytes and not on macrophages. The substantial number of both macrophages and leukocytes could be recognized in the direct vicinity of transplanted MDCs clusters 24 h after injection. At this time point the majority of CD68 expressing cells were surrounding the clusters, and only few macrophages could be observed between the injected cells (Figure 4(a)). At day 3 cells expressing CD68 were totally covering the area of PKH26 clusters (Figure 4(b)). One week after injection the number of macrophages was substantially reduced. In many cases, CD68 colocalized with PKH26 red fluorescence (Figures 4(b) and 4(c)). The presence of CD43 positive cells was the most prominent at day 1 (Figure 5(a)).

### 3.7. Muscle-Derived Cells Are Eliminated from the Injection Site after Autologous Transplantation

**Figure 7:**
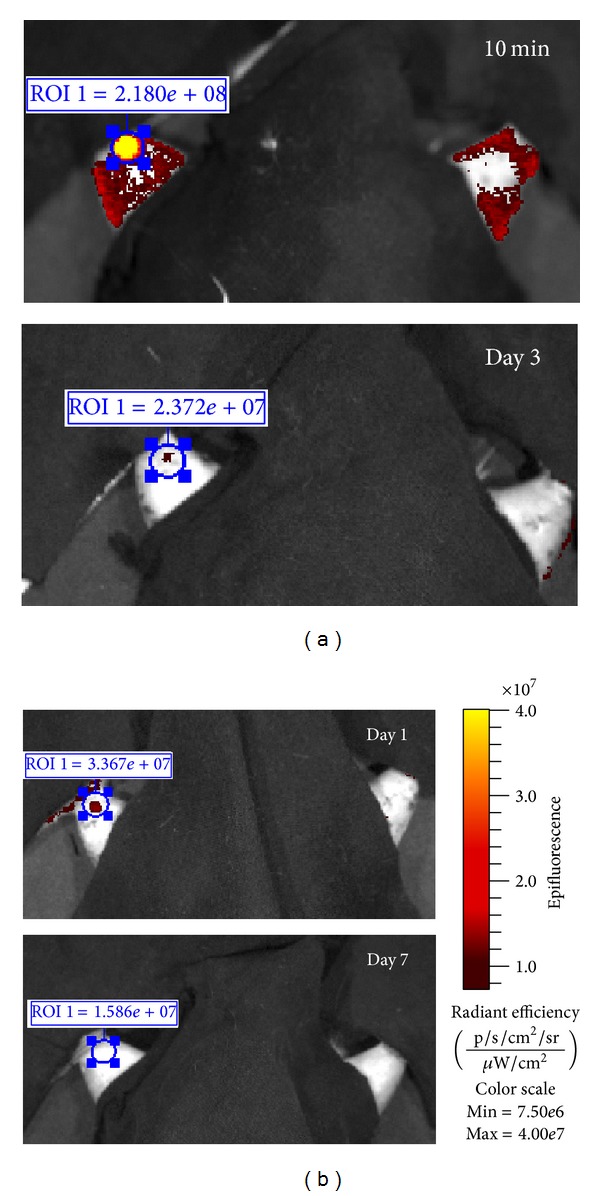
*In vivo* imaging of a representative animal in subsequent time points. 2.5 × 10^5^ DiD-labeled MDCs were injected into the gastrocnemius muscle (limb on the left). Contralateral limb was injected with the equal volume of vehicle and constituted internal control. Optical imaging was carried out at 10 min, days 1, 3, and 7 after the injection. Read-out scale was unified between the subsequent images, as presented on the right panel. Values in blue rectangles indicate total radiant efficiency of DiD-derived signal measured in subsequent time points in equal regions of interest (ROI).

*In vivo* fluorescence imaging revealed the distinct reduction of DiD-derived signal in subsequent time points in all analyzed animals (*n* = 3). Images of representative rat were presented at Figure 7. Vehicle injection did not induce fluorescence in analyzed wavelengths which indicate the specificity of signal detected in the transplanted limb. These results confirm the poor cell survival after intramuscular injection, but for the first time this phenomenon is demonstrated in the same animal in subsequent time points after autologous transplantation.

### 3.8. Survived MDCs Were Able to Fuse with Host Muscle Fibers

**Figure 8:**
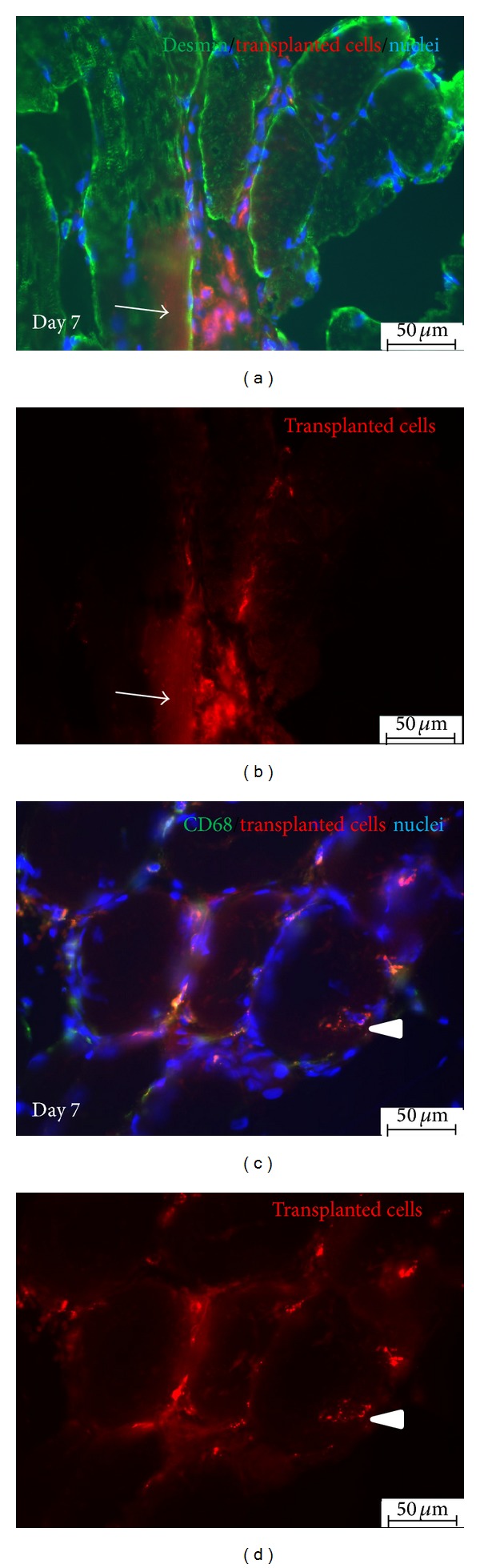
Immunohistochemical staining of samples collected 7 days after autologous MDCs injection. (a and b) PKH26 positive cells (red) can be recognized between and within muscle fibers (arrow), desmin stained with Alexa 488 (green); (c and d) PKH26 positive cells (red) can be recognized in the central position of muscle fiber (arrowhead) indicating the ability to contribute muscle regeneration. Few macrophages (CD68 stained with Alexa 488 (green)) are still present in the site of transplantation. Scale bars: 50 *μ*m.

Labeling of cells* ex vivo* allowed for identification of their fate* in vivo*. At day 7 PKH26 derived fluorescence was observed both in mononuclear cells located between muscle fibers and in the muscle fibers (Figures 8(a) and 8(b)) Moreover, PKH26 positive cells could be recognized in the central position of muscle fibers sections (Figures 8(c) and 8(d)) indicating that transplanted cells possessed ability to participate in the regeneration process.

## 4. Discussion

The poor survival of myoblasts after transplantation is well-known problem which limits the introduction of MTT into the clinic. The elimination of injected myoblasts is massive and rapid. In allogeneic models, 24 h after myogenic cells transplantation only 20% or less of initial cell number can be detected in the area of injection. This amount is further decreasing to 1–5% at days 3–5 after transfer [5, 15, 16]. Autologous way of transplantation does not solve this problem. The process of graft losing seems to be slower; Holzer et al. [7] detected 60% of injected population after 24 h, but the percentage of survived cells diminished to about 10% at day 3 [7]. Herein, we confirm the limited cell survival after autologous intramuscular transplantation by* in vivo* imaging of the same individuals in subsequent time points. The current understanding of such graft failure is not clear. Our previously published results suggest the role of oxidative stress (possibly generated by phagocytic immune cells) in cellular graft elimination after autologous transfer [18]. Therefore the aim of presented herein experiments was to describe the dynamics of acute local inflammatory reaction in response to autologous MDC transplantation.

In our experiments we isolated cells exploiting preplate (pp) technique described by Qu et al. [20]. To reduce the number of fibroblasts in culture we used a subset of cells which adhered to the plastic surface between 24 h and 72 h after seeding. According to preplating schedule presented by Qu et al. [20] we used mixed population of pp3-pp4 cells which mainly consists of satellite cells progeny, myoblasts. Indeed, our results confirm that majority of isolated cells expressed desmin. The average fusion index 3 days after induction of myogenic differentiation amounted to 35%. In comparison, the average fusion index of C2C12 cell line (mice satellite cells) was shown to be about 50% [21]. Therefore, as obtained population still contained some nonmyogenic cells like fibroblasts or fibroadipogenic progenitors, we call it muscle-derived cells rather than myoblasts.

The analysis of samples collected 24 h after transplantation indicated that autologous MDCs induce significant upregulation of* Il-1α* and* Il-1* *β* genes expression in comparison to the group treated with vehicle only (Figure 2). The significantly increased expression of* Il-1*, especially* Il-1* *β* isoform, in response to the intramuscular cell transplantation, was previously reported in several different models: allogeneic transfer of myoblasts into the intact myocardium [5], allogeneic transfer of mesenchymal stem cells (MSCs) into the infarcted myocardium [22], and syngeneic transfer of MSCs into intact skeletal muscle [23]. In the present study we demonstrate for the first time that similar response in regard to* Il-1* *β* expression is observed also after autologous transfer of MDCs. Moreover, we confirmed this finding at the protein level (Figure 3). The evaluation of* Il-1* *β* expression dynamics during the first week after the procedure revealed that the boost of* Il-1* *β* is acute and transient and the expression at day 3 is distinctly lower than at day 1 and it is not any more significantly higher comparing to the VEH group in analogous time point. Suzuki et al. [5] also evaluated the dynamics of cytokines expression in two time points (24 h and 3 days after transfer) after allogeneic myoblasts grafting into intact myocardium and reported the same pattern of changes, peak upregulation at day 1 and downregulation at day 3. As IL-1*β* is the key proinflammatory cytokine associated with the activity of phagocyting immune cells, those data suggest that the dynamic of early inflammatory reaction after MTT is similar in both auto- and allogeneic transplantations. It is worth noting that IL-1 was showed to be important in respect to grafted cell survival in allogeneic model. It was demonstrated that blocking of IL-1 action resulted in increased transplantation efficacy [14, 20]. Our data suggest that the same effect could be obtained in autologous transfer.

In the present study we also characterized early inflammatory infiltration in reaction to MDCs transfer. For this part of experiment cells were labeled with red membrane linker, PKH26, prior to transplantation. This method of labeling was chosen as it is described to be nonimmunogenic in both manufacturer's specification and a published report [24]. Thus it seems to be applicable for studying of immune response. The disadvantage of this cell tracker is substantial diluting during cell divisions which was analyzed in our recently published report [25]. However in short term experiment in which we did not expect intensive cell proliferation membrane linker met our needs. In the tissue samples collected from MDC group at days 1 and 3 the transplanted cells were clearly visible. In those two time points they formed dense, demarcated cell clusters (Figures 4(a) and 4(b)). Very similar appearance was observed after allogeneic transplantation of either myoblasts or muscle-derived stem cells at day 2 after injection [20]. In our experiment, the distribution of grafted cells changed in specimens collected at day 7; the cells were much more diffused throughout the site of injection (Figure 4(c)). The amount of PKH26 positive cells at this time point was distinctly lower comparing to either day 1 or day 3. At day 7 PKH26 derived fluorescence was observed both in mononuclear cells located between muscle fibers and in the muscle fibers (Figure 8(a)). Moreover, PKH26 positive cells could be recognized in the central position of muscle fiber section (Figure 8(b)) which indicated that transplanted cells which survived in the injection site were able to participate in the regeneration process. This finding is consistent with previous reports [7, 20, 26].

The results indicating limited persistence of injected cells at day 7 were confirmed by semiqualitative graft survival analysis using* in vivo* fluorescence imaging (FLI). Although this method has serious limitations because of considerable autofluorescence of the animal tissues, it was previously demonstrated that FLI analysis of myoblasts survival after intramuscular injection can be valid [8]. In our study, we have utilized a spectral unmixing algorithm offered by the Living Image software to alleviate this issue. Using membrane linkers for* in vivo* imaging creates an additional problem; these dyes can be absorbed by phagocytes and therefore impede interpretation of results. However, in our experiment the radiant efficiency of DiD-derived signal decreased markedly in subsequent time points; therefore we can conclude that cells were indeed eliminated from the injection site.

To confirm the presence of immune response to autologous MDCs injection the tissue sections were analyzed in order to identify inflammatory cells. Basic H&E staining revealed that clusters of transplanted cells were infiltrated with polymorphonuclear granulocytes at day 1 (Figure 6(a)). At day 3, the presence of neutrophil in H&E stained sections was not so obvious; most numerous cells within transplanted clusters at this time point were large and mononuclear which could indicate them to be macrophages. To identify the infiltration more precisely the sections were immunostained for CD43—an antigen which is present on majority of leukocytes and not on macrophages. The analysis revealed the typical sequence of events in early innate immune reaction, the rapid infiltration of target area with neutrophils (within first 24 h) and delayed appearance of macrophages with the maximal intensity at day 3. At day 7 the inflammatory reaction was almost completely resolved which is consistent with results of cytokines gene expression analysis. Thus, our results demonstrate for the first time that autologous transplantation of muscle-derived cells induces classical early immune reaction in the site of injection. It can be claimed that the presence of macrophages in the injection site is beneficial for the grafting efficacy. It was previously demonstrated that macrophages enhance or even are required for muscle regeneration [27, 28]. Furthermore, it was shown that coinjection of myoblasts and macrophages improves survival, proliferation, and migration of transplanted muscle precursor cells [9, 29]. However, results regarding coinjection were obtained after either allogeneic or xenogeneic injection into immunodeficient host. Thus, it is difficult to predict if host derived macrophages could also improve transplanted cell survival.

There are two most probable interpretations of innate response appearance. First, the inflammatory cells are activated because the injected cells become immunogenic during extracorporeal manipulations. Second, injected cells die due to other reasons like ischemia, hypoxia, or anoikis and phagocytes infiltrate the graft to clean up dead cells but not to eliminate viable ones. Verification of the mechanism which has predominant importance is crucial for assessment of future strategies for autologous MTT efficacy improvement. Our previously published data demonstrated that cells with increased resistance to oxidative stress survive better after autologous intramuscular transplantation. Similar findings were presented before by Urish et al. [26] on allogeneic model of transplantation. As the production of reactive oxygen species is associated with phagocyting activity, thus the importance of resistance to oxidative stress supports the hypothesis that inflammation is the cause and not the consequence of grafted cell death. The role of inflammation in myoblasts elimination after autologous administration was also shown by Ito et al. [30]. The authors demonstrated that strong systemic immunosuppression improved the efficacy of transplantation in nondystrophic dogs. On the other hand some reports suggest that neutrophils and macrophages are not responsible for early death of donor myoblasts even in allogeneic model [15, 16] and thus support the theory that cells die due to other reasons and inflammation is only the secondary consequence of this death. As the two potential mechanisms are not exceptive, it is possible that only complex protective approach will be able to effectively prevent myoblasts' death after transplantation.

To conclude, presented data demonstrate that autologous intracellular MDCs transplantation induces classical early immune reaction in the site of injection. These results, when considered with previously published reports, suggest that innate inflammatory response can contribute to limited survival of theoretically nonimmunogenic autologous cellular graft.
